# Evaluating Dermal Bone as a Novel Source of Endocrine Information in Ninespine and Threespine Stickleback Fish

**DOI:** 10.1093/iob/obad007

**Published:** 2023-02-28

**Authors:** D Dillon, P E Witten, C L Buck

**Affiliations:** Department of Biological Sciences, Northern Arizona University, 617 S. Beaver St., Flagstaff, AZ 86011, USA; Research Group Evolutionary Developmental Biology, Ghent University, Ledeganckstraat 35, 9000 Ghent, Belgium; Department of Biological Sciences, Northern Arizona University, 617 S. Beaver St., Flagstaff, AZ 86011, USA

## Abstract

Monitoring the physiology of small aquatic and marine teleost fish presents challenges. Blood samples, often the first choice for endocrinologists, can be difficult or even impossible to obtain and alternative matrices currently used for hormone analyses do not occur in fishes (e.g., hair, feathers etc.) or are not easily collected from small aquatic organisms (e.g., urine and feces). Some teleosts, however, have enlarged bony dermal elements that possibly accumulate and store steroid hormones in physiological relevant concentrations. Both threespine stickleback (*Gasterosteus aculeatus*) and ninespine stickleback (*Pungitius pungitius*) have a series of external, lateral bony plates, dorsal spines, and a pair of pelvic spines attached to the pelvic girdle. We investigated if cortisol, the primary circulating glucocorticoid in teleosts, could be extracted from stickleback dermal bone and quantified using a commercially available enzyme immunoassay (EIA). We successfully validated a cortisol EIA for dermal bone extracts, determined that cortisol was detectable in both species, and found that dermal bone cortisol levels significantly correlated with cortisol levels in whole body homogenate. Ninespine stickleback had significantly higher dermal bone cortisol concentrations than threespine stickleback and female threespine stickleback tended to have over twice the mean dermal bone cortisol concentration than males. Because both stickleback species are widely used for ecotoxicological studies, using dermal bone as a source of endocrine information, while leaving the body for contaminant, genomic, histological, and stable isotope analyses, could be a powerful and parsimonious tool. Further investigation and physiological validations are necessary to fully understand the utility of this new sample matrix.

## Abstract (es)

Spanish (translation provided by Luis C. Beltran)

El monitoreo de la fisiología de pequeños peces teleósteos acuáticos y marinos presenta desafíos. Las muestras de sangre, a menudo la primera opción para los endocrinólogos, pueden ser difíciles o incluso imposibles de obtener y las matrices alternativas que se utilizan actualmente para los análisis hormonales no son aplicables en peces (p. ej., pelo, plumas, etc.) o no se obtienen fácilmente de pequeños organismos acuáticos (p. ej., orina y heces). Sin embargo, algunos teleósteos tienen elementos dérmicos óseos agrandados que posiblemente acumulan y almacenan hormonas esteroides en concentraciones fisiológicas relevantes. Tanto el espinoso de tres espinas (*Gasterosteus aculeatus*) como el espinoso de nueve espinas (*Pungitius pungitius*) tienen una serie de placas óseas laterales externas, espinas dorsales y un par de espinas pélvicas unidas a la cintura pélvica. Nosotros investigamos si el cortisol, el principal glucocorticoide circulante en los teleósteos, podría extraerse del hueso dérmico del espinoso y cuantificarse mediante un inmunoensayo enzimático (EIA, por sus siglas en inglés) disponible comercialmente. Validamos con éxito un EIA de cortisol para extractos óseos dérmicos, determinamos que el cortisol era detectable en ambas especies, y descubrimos que los niveles de cortisol óseo dérmico se correlacionaron significativamente con los niveles de cortisol en el cuerpo entero homogeneizado. El espinoso de nueve espinas tenía concentraciones de cortisol óseo dérmico significativamente más altas que el espinoso de tres espinas y el espinoso de tres espinas hembra tendía a tener más del doble de la concentración media de cortisol óseo dérmico que los machos. Debido a que ambas especies de espinosos se usan ampliamente para estudios ecotoxicológicos, el uso del hueso dérmico como fuente de información endocrina, mientras se deja el cuerpo para análisis de contaminantes, genómicos, histológicos e isótopos estables, podría ser una herramienta poderosa y parsimoniosa. Se necesitan más investigaciones y validaciones fisiológicas para comprender completamente la utilidad de esta nueva matriz de muestra.

## Abstract (pt)

Portuguese (translation provided by Jonathan M. Branco)

Monitorar a fisiologia de pequenos peixes teleósteos aquáticos e marinhos apresenta desafios. Amostras de sangue, muitas vezes a primeira escolha para endocrinologistas, podem ser difíceis ou mesmo impossíveis de obter e matrizes alternativas atualmente usadas para análises hormonais não ocorrem em peixes (e.g., pelos, penas etc.) ou não são facilmente coletadas em pequenos organismos aquáticos (e.g., urina e fezes). Alguns teleósteos, no entanto, possuem elementos dérmicos ósseos ampliados que possivelmente acumulam e armazenam hormônios esteroides em concentrações fisiológicas relevantes. Ambos *Gasterosteus aculeatus* e *Pungitius pungitius* apresentam uma série de Placas ósseas externas laterais, espinhos dorsais e um par de espinhos pélvicos ligados à cintura pélvica. Investigamos se o cortisol, o principal glicocorticoide circulante em teleósteos, poderia ser extraído do osso dérmico de *G. aculeatus* e *P. pungitius* e quantificado usando um imunoensaio enzimático (EIA) comercialmente disponível. Validamos com sucesso um EIA de cortisol para extratos de ósseos dérmicos, determinamos que o cortisol era detectável em ambas as espécies e descobrimos o cortisol ósseo dérmico em níveis significativamente correlacionados com os níveis de cortisol em todo o corpo homogeneizado. *P. pungitius* apresentou concentrações de cortisol ósseo dérmico significativamente mais altas do que *G. aculeatus* e fêmeas de *G. aculeatus* tenderam a ter mais que o dobro da concentração média de cortisol ósseo dérmico de machos. Como ambas as espécies são amplamente utilizadas para estudos ecotoxicológicos, usando o osso dérmico como fonte de informação endócrina, deixando o corpo para análises de contaminantes, genômicas, histológicas e de isótopos estáveis, pode ser uma ferramenta poderosa e parcimoniosa. Mais investigações e validações fisiológicas são necessários para entender completamente a utilidade desta nova matriz de amostra.

## Introduction

Vertebrates exposed to environmental stressors express a wide range of physiological changes broadly thought to enhance survivorship (Sapolsky et al. 2000; Reeder and Kramer 2005; Wingfield and Romero 2011; Romero and Wingfield 2016). These changes collectively comprise the adaptive stress response and are largely modulated by the hypothalamic–pituitary–adrenal axis (HPA; avian and non-avian reptiles, mammals, and amphibians) or the hypothalamic–pituitary–interrenal axis (HPI) in teleost fish. Glucocorticoids (cortisol and/or corticosterone), synthesized and secreted by the adrenal glands or interrenal cells in response to stressful stimuli, modulate energy allocation to survive the stress event(s) or recover following exposure to stressful stimuli (Reeder and Kramer 2005). Avian and non-avian reptiles, amphibians and rodents primarily secrete corticosterone, while cortisol is the dominant glucocorticoid secreted by teleosts and most mammals.

Various vertebrate tissues and biological substrates can be sampled for glucocorticoids, including serum/plasma, feces, urine, saliva, milk, eggs, blubber, cerumen, respiratory vapor, skin mucus, bones, teeth, and a growing number of keratinous tissues including hair, feather, baleen, shed skin, whiskers, claws, spines, and scales (representative references listed in Table 1, excluding those discussing serum/plasma and feces). Each of these sample types represents a specific timeframe over which the glucocorticoids are incorporated (minutes to days to weeks to years) and thus can potentially resolve questions about the latency and timeline of the adaptive stress response (Sheriff et al. 2010; Cook 2012; Kersey and Dehnhard 2014; Palme 2019).

**Table 1:** Representative references describing unique sample types to obtain endocrine information across vertebrate classes

Class	Sample type	Reference
Amphibia	Urine	Narayan et al. 2010
		Narayan 2013
Aves	Eggs	Eriksen et al. 2003
	Feathers	Bortolotti et al. 2008
Mammalia	Baleen	Fernández Ajó et al. 2018, 2020
		Hunt et al. 2014b
	Blubber	Mansour et al. 2002
	Bones	Charapata et al. 2018
		Yarrow et al. 2010
	Cerumen	Trumble et al. 2013
	Claws	Veronesi et al. 2015
		Karpovich et al. 2020
		Crain et al. 2021
	Hair	Koren et al. 2002
	Hooves	Comin et al. 2014
	Milk	Butler and Bordes 1980
		Reviewed in Stead et al. 2022
	Respiratory vapor	Hunt et al. 2014a
	Saliva	Reviewed in Clegg and Millson 2021
	Scales	Blecher et al. 2021
	Spines	Fabio Braga et al. 2022
	Teeth	Hudson et al. 2021
	Urine	Reviewed in Clegg and Millson 2021
	Whiskers	Lübcker et al. 2020
		Karpovich et al. 2019
Osteichthyes	Eggs	Reviewed in Sopinka et al. 2017
	Mucus	Simontacchi et al. 2008
		Carbajal et al. 2019a
	Opercula	Charapata et al. 2022
	Scales	Aerts et al. 2015
		Carbajal et al. 2018, 2019a
		Laberge et al. 2019
Reptilia	Claws	Baxter-Gilbert et al. 2014
		Matas et al. 2016
	Eggs	Conley et al. 1997
	Shed skin	Berkvens et al. 2013
		Zena et al. 2021

Biological “hard parts,” such as keratinous tissues and bone, integrate hormones over the timeframe in which they are grown. While keratinous tissues are metabolically inert after growth, bones experience continuous turnover. Both sample types, however, can provide a record of physiological responses to environmental conditions. For example, Fairhurst and colleagues (2015) demonstrated that feathers from museum specimens dating back to 1859 could be used to examine historical patterns of glucocorticoid secretion and stable isotope incorporation. Baleen, the keratinous feeding apparatus of mysticete whales, captures the hormone and toxicant profiles of an individual over the duration of plate growth, encompassing a year to a decade or more, depending on the species and age of the individual (Hunt et al. 2014, 2016, 2017a, 2018; Rolland et al. 2019; Fernández Ajó et al. 2020; Lowe et al. 2022). Deposited hormones appear to persist in the keratinous matrix over centuries or longer. Kellner et al. (2022) found elevated cortisol in ancient (450–1000 years old) human hair from individuals in the month before death. Similarly, bone has been shown to accumulate and maintain steroid hormones; progesterone, testosterone, cortisol, and estradiol were quantified in modern, historical and even 3500 years old archeological walrus bone (Charapata et al. 2018). The authors postulate that the concentrations found in walrus cortical bone represented an average of levels incorporated over a 10- to 20-year period. Additionally, and most relevant to the current study, Charapata et al. (2022) extracted steroid hormones from rockfish opercula and obtained valuable insight about lifetime reproduction and stress. These findings extend those of Yarrow et al. (2010), who described a method for extraction and quantification of sex steroids from the bones of 4-month-old male rats. Additionally, teeth from four marine mammal species have been demonstrated to be a novel source of endocrine information (Hudson et al. 2021). Interestingly, the authors detected reproductive hormones, corticosterone and triiodothyronine (T3), but were not able to detect cortisol in any of the tooth extracts they tested, even though cortisol is generally thought to be the primary circulating glucocorticoid in most mammals.

Until recently, there were few sample types available to study the endocrine profiles of aquatic and marine organisms, particularly if one was interested in a temporal window beyond the immediate snapshot offered by plasma. Baleen, blubber, feces, scales, cerumen, whiskers, claws, opercula, and teeth have been the best explored matrices from aquatic and marine organisms that allow for a retrospective analysis of an individual's endocrine function (see Table 1). Modern and historic tissues could be used to address questions related to global climate change, local extreme weather events, ecosystem disruptions or shifts in toxicant exposure, among others. It is clear that these perturbations have an outsized impact on the world's aquatic and marine environments and more tools are needed to study the effects on aquatic organisms (Brander 2007; von Hippel et al. 2018; Cheung and Frölicher 2020; Servili et al. 2020).

Chronic exposure to aquatic pollutants and/or toxicants has been shown to negatively impact teleosts, partly through disruption of the HPI axis (Hontela et al. 1992; Arukwe 2001; Scott and Sloman 2004; Pankhurst 2011; Ismail et al. 2017). Assessing the effects of chronic activation of the HPI axis on a fish can be a challenge, especially if the animal is small and obtaining blood samples of sufficient quantity is neither feasible nor desired (Sadoul and Geffroy 2019). Pairing an integrated measure of HPI activation with toxicant levels in organs, muscle, etc., from the same individual could be extremely valuable, especially if the exposure and biological response represents the same time-period. Carbajal and colleagues (2019b) explored this by measuring cortisol in scales and plasma and analyzing muscle samples for perfluoroalkyl and polyfluoroalkyl substances (PFAS) in fish collected from a polluted river and a reference site. Additionally, baleen has been used to investigate mercury exposure and possible correlation with hormone levels in individual humpback whales (*Megaptera novaeangliae*) (Lowe et al. 2022). Lastly, teeth have long been recognized for their capacity to incorporate and store contaminants and thus can be used to reconstruct an individual's history of contaminant exposure (Sharon 1988; Bercovitz and Laufer 1993; Eide et al. 1993; Tvinnereim et al. 2000).

Threespine stickleback (*Gasterosteus aculeatus*) and ninespine stickleback (*Pungitius pungitius*) are small, advanced teleosts distributed throughout the northern hemisphere (Wootton 1976; Hardy 1978). Stickleback are widely used in ecotoxicological studies in both field and laboratory investigations because of their abundance across a wide range of aquatic and marine habitats, ease of collection and rearing, and resilience to toxicant exposure (Petersen et al. 2015, 2022; von Hippel et al. 2016, 2018; Gardell et al. 2017). Additionally, the genomes of both species are sequenced and well-studied (Peichel et al. 2001, 2017; Nelson and Cresko 2018; Varadharajan et al. 2019). Given that the endocrine system is highly conserved among vertebrates (Baker 2003), this combination of traits makes these fish a good model species to investigate questions of organismal responses to challenges faced in an aquatic environment (Katsiadaki et al. 2007; von Hippel et al. 2016).

A feature common to both ninespine and threespine stickleback is presence of distinct dermal skeletal elements comprising a series of external, lateral bony plates, dorsal spines, and a pair of pelvic spines attached to the pelvic girdle (Reimchen 1983; Song et al. 2010; Lees et al. 2012). The biology of dermal bone and its formation has been extensively researched (Sire and Huysseune 2003; Sire et al. 2009). Briefly, dermal bones are formed in the dermis without a cartilaginous template, a process known as intramembranous bone formation. Elements that belong to the dermal skeleton are considered derivatives of ancestral odontodes and include teeth, ganoid and elasmoid scales, scutes, bony fish spines, and dermal fin rays (Sire and Huysseune 2003).

In contrast to the dermal skeleton, several components of the endoskeleton are preformed as cartilage and are subsequently replaced by bone, a process known as endochondral bone formation (Witten et al. 2017). Advanced teleosts (i.e., stickleback) have acellular bone. That is, osteoblasts and osteoclasts are present, but osteocytes have been lost in all parts of the skeleton. Consequently, and different from basal osteichthyans and tetrapods, dermal bones are acellular. Further, teleost bone marrow contains no hematopoietic tissue. Hematopoiesis takes place in the head kidney and bone marrow spaces are filled with adipose tissue (Witten and Huysseune 2007, 2009). Despite the lack of osteocytes, the dermal skeleton continuously grows throughout the life of the stickleback and has the capacity to regenerate (Reimchen 1988, 2021).

Because teleosts and many other vertebrates can integrate steroid hormones and T3 into various biologically inert tissues, and hormones have been extracted and quantified from modern and ancient bones and teeth, we hypothesized that circulating hormones are deposited and quantifiable in stickleback dermal bone elements. We previously found detectable levels of corticosterone in ninespine stickleback dermal bone as part of a larger investigation of keratinase enzyme digestion prior to extraction to improve steroid hormone recovery (unpublished data; Dillon et al. 2021). With the current study, we set out to develop and optimize a hormone extraction protocol for ninespine and threespine stickleback dermal bone, validate a commercially available EIA for cortisol for use with the extracts, and compare dermal bone cortisol levels to that found in body homogenate.

## Materials and methods

### Sample collection and preparation

Ninespine stickleback were collected from Troutman Lake on St. Lawrence Island, Alaska (63.77317°N, 171.70987°W) in July 2018 and anadromous threespine stickleback were collected from Rabbit Slough, Alaska (61.5595°N, 149.2583°W) in July 2012. Both species were collected using unbaited minnow traps and euthanized using an overdose of neutralized MS-222. Individual fish were placed into plastic bags, placed on ice until returned to the laboratory and then stored at −80°C. In 2021 and 2022, 40 ninespine stickleback (20M, 20F) and 10 threespine stickleback (5M, 5F) were weighed on a digital balance (±0.0001 g; Ohaus Explorer Pro EP214C, Pine Brook, NJ, USA). All fish used in this study were reproductively mature adults, as determined by gonads and external phenotype (nuptial coloring and enlarged testes in males, enlarged abdomens and ovaries in females). All dermal bone was removed with forceps, any visible skin remaining was removed, and the sample was lightly sprayed with 70% ethanol and blotted dry before being placed in to a 12 × 75 mm borosilicate glass tube. Dermal bone samples were freeze-dried under vacuum (Labconco FreeZone Freeze Dry System with Stoppering Tray Dryer; LabConco, Kansas City, MO, USA) and then weighed again (Table 2; ±0.0001 g; Ohaus Explorer Pro EP214C, Pine Brook, NJ, USA). Each sample tube received 4 mL absolute methanol (Dillon et al. 2021; Fernández Ajó et al. 2022) and was shaken overnight (Bortolotti et al. 2008) on a multitube vortexer at room temperature (Glas-Col Large Capacity Mixer, speed set on 65; Glas-Col, Terre Haute, IN, USA). Tubes were centrifuged for 15 min at 1056 g, the supernatant (3.8 mL) was collected in a clean 12 × 75 mm borosilicate glass tube and dried in a ThermoSavant SpeedVac Concentrator (model SDP121P; Thermo Fisher Scientific, Waltham, MA, USA) at 35°C and stored at −80°C.

**Table 2:** Mean and range of sample masses (mg) for ninespine and threespine stickleback dermal bone and body homogenate

	Mass (mg)		
	Mean	Min	Max
**Ninespine**			
Dermal bone	12.8	6.9	24.8
Body homogenate	13.3	7.4	25.5
**Threespine**			
Dermal bone	181.1	72.9	232.3
Body homogenate	183.2	83.9	236.2

#### Body homogenate preparation

After dermal bones were removed, each body was freeze-dried under vacuum (Labconco FreeZone Freeze Dry System with Stoppering Tray Dryer; LabConco, Kansas City, MO, USA), chopped into 1 cm pieces and transferred to 1.5 mL stainless steel vials (BioSpec Products Inc., Bartlesville, Oklahoma). Two stainless steel beads (3.2 mm) were added to the vials before they were capped with a rubber stopper and then placed in a Mini-BeadBeater (model 112011 BioSpec Products Inc., Bartlesville, OK, USA) at 275 oscillations per min for 3 min. A mass of body homogenate powder equal to the mass of the dermal bones was weighed (Table 2) and transferred to a 13 × 100 mm (ninespine) or 16 × 100 mm (threespine) borosilicate glass tube. Each tube received 4 mL ethyl acetate (modified from Arbor Assays Steroid Solid Extraction Protocol, https://www.arborassays.com/wp-content/uploads/2022/02/Steroid-Solid-Extraction-190222.pdf) before being capped and shaken for 1 h on a multi-tube vortexer at room temperature (Glas-Col Large Capacity Mixer, speed set on 65; Glas-Col, Terre Haute, IN, USA). Tubes were centrifuged for 10 min at 1056 g and the supernatant was collected in a clean borosilicate glass tube. The pellet was extracted twice more with 2 mL ethyl acetate (30 min shake, 10 min centrifuge), the supernatants combined and dried in a ThermoSavant SpeedVac Concentrator (model SDP121P; Thermo Fisher Scientific, Waltham, MA, USA) at 35°C and stored at −80°C.

### Assay

One day before assay, all extracts were resuspended in 0.5 mL assay buffer (buffer X065, Arbor Assays), vortexed for 1 h and then stored at 4°C overnight. Threespine stickleback dermal bone extracts were diluted 1:2 with assay buffer and ninespine stickleback dermal bone extracts were diluted 1:4 (dilution factors were determined during assay validation; see Section “Assay validations” for details) and then assayed for cortisol using a commercially available enzyme immunoassay kit (cat #K003; Arbor Assays, Ann Arbor, MI, USA). Assays were conducted according to the manufacturer's protocol with a full standard curve (50–3200 pg/mL) and a lab control of known concentration. All standards, samples, and controls were assayed in duplicate. Any sample duplicates which exceeded 10% coefficient of variation (CV) were re-assayed (no samples were rerun for this reason). If samples were outside the range of the standard curve, the dilution factor was adjusted and the sample was re-assayed. Three ninespine dermal bone samples, two ninespine body samples, and seven threespine dermal bone samples were diluted 1:20; three threespine dermal bone samples were diluted 1:100 and two threespine body samples were diluted 1:60. Interassay variation (14.1%) was calculated using the values of the known concentration control run on every plate and intraassay variation (2.4%) represented the average of all unknown CVs over all plates.

### Assay validations

Tests of parallelism and accuracy were performed with dermal bone extracts from both species to assess assay performance. Parallelism involves assaying serial dilutions (1:2–1:128) of pooled dermal bone extracts (pool made from aliquots from each sample for each species) alongside the assay kit's standard curve in order to determine if the assay antibody has good binding affinity for the hormone of interest in the sample, in this case, cortisol. Additionally, we used the test of parallelism to determine the appropriate dilution for each species and treatment that would best approximate 50% binding (i.e., the most precise portion of the standard curve). If the slopes of the sample dilution and standard curves were determined not to be statistically different, we concluded the sample behaved immunologically similar to the standard curve and could be measured proportionally. For the accuracy test, each standard (50, 100, 200, 400, 800, 1600, and 3200 pg/mL) was spiked with sample pool and assayed, thus measuring the potential interference of other molecules in the extract (“matrix effect,” Grotjan and Keel, 1996). We define acceptable accuracy (i.e., little to no interference or matrix effect) as a line with an R^2^ > 0.95 and a slope between 0.7 and 1.3 (ideal slope = 1) (Hunt et al. 2017b, 2018; Fernández Ajó et al. 2018; Dillon et al. 2021).

### Statistical approach

For parallelism, we plotted the % binding (%B/B0) vs log_10_ (relative dose) and the linear portions of both curves (sample serial dilutions and standards) and compared slopes using an F-test. We used a linear regression of observed vs expected dose to assess accuracy. Measured cortisol concentrations were not normally distributed (Shapiro–Wilk test; ninespine bone W = 0.5469, *P* = 0.0001; ninespine body W = 0.4965, *P* = 0.0001; threespine bone W = 0.7952, *P* = 0.0127; threespine body W = 0.5202, *P* = 0.0001). Due to this, and the sample size disparity, a Mann–Whitney test was used to assess differences between species and sex differences within species. The relationship between dermal bone cortisol and body homogenate cortisol was assessed using a Spearman correlation. All analyses were performed using Graphpad Prism 9 (Graphpad Software, San Diego, CA, USA). Data are plotted as medians ± interquartile range (IQR), and differences were considered significant at *P* < 0.05.

## Results

**Fig. 1:**
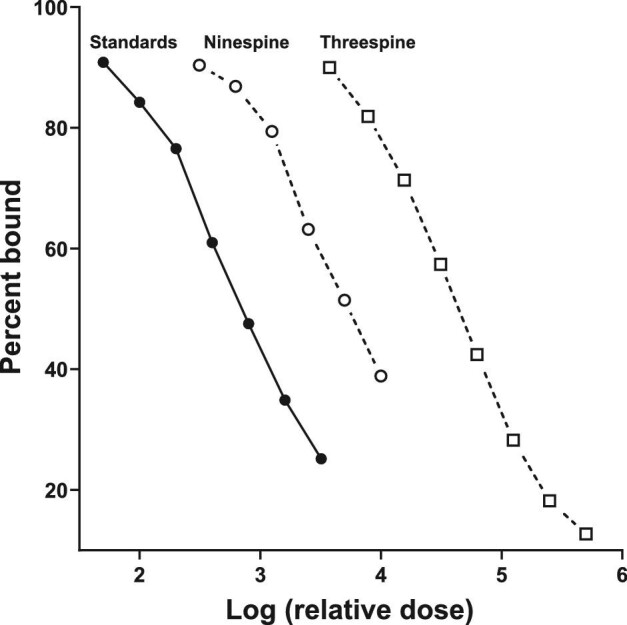
Serially diluted dermal bone extracts from ninespine stickleback (open circles) and threespine stickleback (open squares) are parallel to/not significantly different (all F-test *P*-values > 0.1) from the cortisol standard curve (closed circles).

**Fig. 2:**
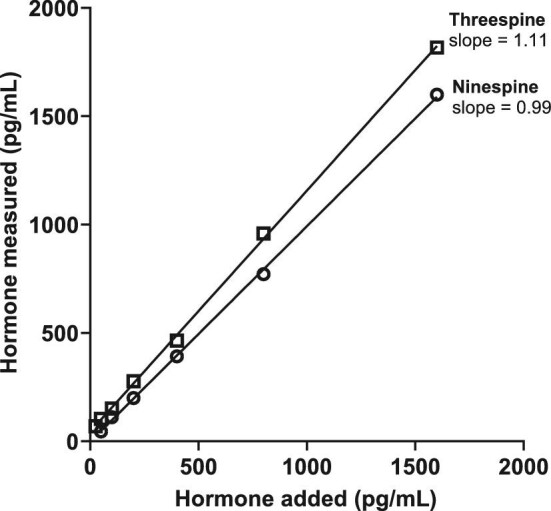
Results of assay accuracy (“matrix effect”) tests for dermal bone extracts from ninespine stickleback (open circles) and threespine stickleback (open squares) spiked with known amounts of hormone. Both extracts had slopes between 0.7 and 1.3 (ideal = 1), indicating hormone can be measured with good mathematical accuracy across a range of concentrations.

**Fig. 3:**
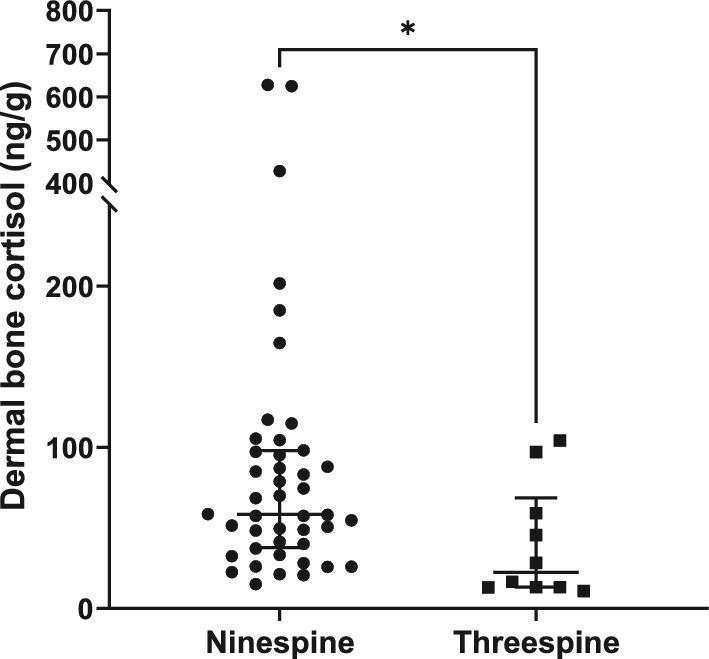
Immunoreactive cortisol (ng cortisol/g dermal bone) is present in all ninespine stickleback (n = 40) and threespine stickleback (n = 10) dermal bone extracts tested and the two species differ significantly (Mann–Whitney U = 111, *P* = 0.0140). Bars represent the median and IQR; axis break at 250 ng/g to better visualize both data sets.

**Fig. 4:**
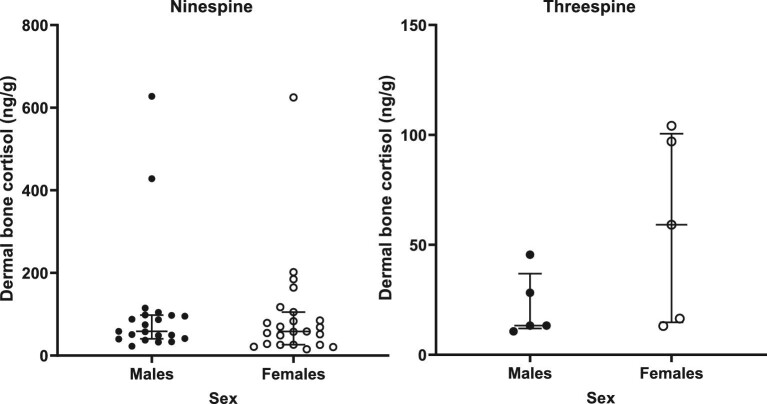
Female threespine stickleback (right panel; open circles) trended toward higher dermal bone cortisol than males (closed circles). No significant sex differences were found in ninespine stickleback (left panel). Bars represent the median and IQR.

**Fig. 5:**
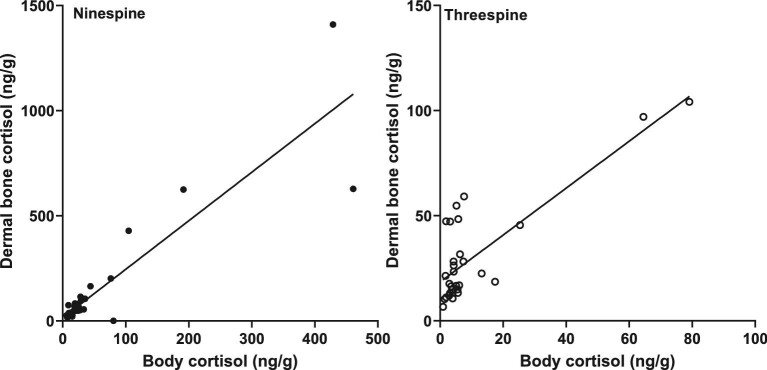
Dermal bone cortisol concentration is highly correlated with body homogenate cortisol concentration in both ninespine (Spearman r = 0.7269, *P* < 0.0001; n = 40) and threespine stickleback (Spearman r = 0.8667, *P* = 0.0022; n = 10).

Immunoreactive cortisol was detectable in all dermal bone extracts from ninespine and threespine stickleback, with acceptable parallelism to the standard curve (Fig. 1, Table 3) and acceptable accuracy (Fig. 2, Table 3). Median concentration of immunoreactive cortisol (ng cortisol/g dermal bone; interquartile range (IQR)) for ninespine dermal bone extracts was 58.3 ng/g (IQR = 37.8–97.9) and 22.3 ng/g (IQR = 13.2–68.6) for threespine extracts (Fig. 3). The difference between species was significant (Mann–Whitney U = 111, *P* = 0.0140). There was no significant difference in median hormone content between male and female ninespine (Mann–Whitney U = 220, *P* = 0.6250; Fig. 4) or threespine stickleback (Mann Whitney U = 6, *P* = 0.222). However, female threespine stickleback tended to have nearly five times the median dermal bone cortisol concentration of males (59.2 vs 13.3 ng/g; Fig. 4); this difference was not statistically significant, most likely due to the small sample size and female individual variation (IQR = 14.8–100.6 ng/g). Dermal bone and body homogenate cortisol were highly correlated within individual ninespine (Spearman r = 0.7269, *P* < 0.0001; Fig. 5) and threespine (Spearman r = 0.8667, *P* = 0.0022; Fig. 5) stickleback fish. No significant relationship was found between body mass and cortisol levels in either matrix, in either species.

**Table 3:** F-test (parallelism) and linear regression (accuracy) results of cortisol immunoassay validation tests for extracts of ninespine and threespine stickleback dermal bone samples

Species	Parallelism	Accuracy
Ninespine	F_1,9_ = 1.643 P = 0.2320	Slope = 0.9944 r^2^ = 0.9995
Threespine	F_1,11_ = 0.2026 P = 0.6614	Slope = 1.112 r^2^ = 0.9994

Each parallelism test compared slopes of the linear portion of the binding curves of assay standards vs serial diluted extract. For accuracy, slope of observed vs expected dose (acceptable range of slope = 0.7–1.3) and coefficient of determination (r^2^; ideally close to 1.00) of the linear regression line are shown.

## Discussion

We explored the use of ninespine and threespine stickleback dermal bone as a source of endocrine data and performed the initial validations of parallelism and accuracy to assess whether cortisol could be reliably quantified in this novel matrix. Using a simple methanol extraction, we found highly detectable levels of cortisol in both ninespine and threespine stickleback dermal bone and the values correlated with cortisol levels found in body homogenate. To the best of our knowledge, this is the first exploration and assay validation of any hormone extracted from dermal bone.

### Cortisol in teleost fish

The function of cortisol in teleost fish is complex; it is not simply a mediator of the stress response, it also modulates osmoregulation, growth, immune function, and reproduction, among other behaviors and physiologies (Mommsen et al. 1999; Pankhurst 2011; Charapata et al. 2022). Correspondingly, all dermal bone samples we tested had detectable cortisol levels irrespective of species or sex, and dermal bone cortisol was significantly correlated with body homogenate cortisol. However, we found interesting species and sex differences in dermal bone cortisol concentration.

### Species differences

Ninespine stickleback had over twice the median dermal bone cortisol content than threespine stickleback. The relatively high levels we measure in ninespine stickleback could be due to their site of collection. These animals were collected from a site known to be contaminated with various toxicants (Byrne et al. 2015, 2017; von Hippel et al. 2018; Zheng et al. 2020) and chronic exposure could lead to high and sustained levels of plasma cortisol, which would be then accumulated in dermal bone. However, it is known that cortisol levels can either increase or decrease in response to toxicant exposure and the direction of change is dependent upon species, toxicant, and exposure duration (Romero and Wingfield 2016; Thang et al. 2017). Because we do not know the expected direction of change in cortisol secretion upon contaminant exposure, we can only speculate that contaminant exposure could be increasing cortisol in these ninespine stickleback. The difference in cortisol concentration may simply be a species difference, and thus assaying dermal bone cortisol from ninespine stickleback collected at a pristine site could elucidate whether the chronic toxicant exposure consistently results high measured cortisol levels or if there are other reasons to explain the stark interspecies difference.

### Sex differences

We investigated sex effects in dermal bone cortisol content for each species and found that female threespine stickleback had about five times the median dermal bone cortisol as males and that there were no significant sex differences in ninespine stickleback. The finding of high cortisol in the dermal bone of female threespine stickleback is consistent with the fact that these animals were sampled during the peak of their reproductive season (Wootton 1976; Karve et al. 2013) and that reproduction in female threespine stickleback is heavily influenced by cortisol (Graham et al. 2018). Specifically, growth is mediated through an interaction between cortisol and growth hormone (reviewed in Mommsen et al. 1999), and the likelihood of reproducing, and subsequent clutch size, is primarily size-dependent (Baker et al. 2015). Moreover, cortisol modulates mobilization of the glucose needed to satisfy the huge energy requirements of reproduction (Mommsen et al. 1999). Cortisol, in concert with prolactin, also regulates many aspects of anadromy, including facilitating physiological changes needed to withstand transitions between hypotonic freshwater and hypertonic marine environments (Audet et al. 1985), stimulating growth, and even acting on the brain to potentially enhance memory of a natal stream (McCormick 2009). Although the ninespine stickleback were reproductively mature at the time of capture (Wootton 1976), they were collected at a higher latitude where the ice on the lake had only recently thawed. Quite likely, these ninespine stickleback were not reproductive for as long as were the threespine stickleback harvested at a lower latitude and thus we would not expect to see the “signature” of high and sustained cortisol levels in the dermal bone at that point.

### Limitations and future work

While the mechanism and timeframe of hormone deposition into stickleback dermal bone is not clear, we can speculate based on what is known about the composition and growth pattern of this tissue. In threespine stickleback, pelvic spines appear as soon as 9 days after hatching, with the first lateral plates appearing 60 days post-hatch (Swarup 1958). Since these elements are continuously growing and can regenerate if damaged (Reimchen 1988, 2021), the measured hormone could represent a lifetime average of the fish or just part of the lifetime. The source of the measured hormone in the dermal bone adds an additional facet of complexity; it could be contained within the surface cells, the bone matrix itself, or the adipose tissue inside the bone (if present). It is possible that there is turnover in this adipose tissue and that the hormone levels better reflect the timeframe of the turnover, rather than an average lifetime load. Also, the significant correlation between individual dermal bone cortisol and body homogenate cortisol in both species is compelling and warrants further investigation. It is unknown whether other hormones would show as strong of a relationship. Incorporation and turnover rates could be experimentally tested using lab-reared stickleback, similar to what was demonstrated with scale cortisol in common carp (*Cyprinus carpio L*.; Aerts et al. 2015).

## Conclusions

Here we present a new method to gain endocrine information while leaving the rest of the body of the fish for other analyses. Both threespine and ninespine stickleback are important model species in ecotoxicology studies (Katsiadaki et al. 2002, 2005, 2007; Hahlbeck et al. 2004; Hogan et al. 2008; Sanchez et al. 2008; Scholz and Mayer 2008; Sebire et al. 2008; Pottinger et al. 2013; von Hippel et al. 2016, 2018; Adams et al. 2019; Zheng et al. 2020; Petersen et al. 2022) and thus being able to use an individual fish for physiological, genomic, isotope and toxicological analyses is powerful and parsimonious. These fish are generally too small to obtain sufficient plasma for endocrine studies and whole body homogenate, as our laboratory has used in the past for endocrine assays, not only presents challenges with interpretation but also does not leave the body available for other analyses (Furin et al. 2015; Petersen et al. 2015; Gardell et al. 2017).

Having an integrated measure of an individual fish's endocrine function (dermal bone) coupled with toxicological or histological data (body) is a powerful tool to assess causality (e.g., does a toxicant elicit an endocrine response?). We predict steroid and other hormones (e.g., T3) will be detectable as well, given their presence in keratinous tissues, bone and teeth (see references in Table 1). It will be important to conduct the necessary assay validations for any additional hormones and investigate whether the same relationship between dermal bone and body homogenate hormone levels exists. Conducting laboratory experiments with captive ninespine and threespine stickleback to determine hormone incorporation and possible turnover rates of dermal bone is an important next step.

## Data Availability

Data available on request.
